# Innovating under different competitive strategies: The impact of R&D on risk and return in dynamic environments

**DOI:** 10.1371/journal.pone.0325130

**Published:** 2025-06-05

**Authors:** Drini Morina, Henning Lucas, Stefanie Heiden

**Affiliations:** Institute for Innovation Research, Technology Management and Entrepreneurship, Leibniz University Hannover, Hannover, Germany; University of Klagenfurt, AUSTRIA

## Abstract

The prevailing narrative in the management literature views R&D as a high-risk, high-return activity. Although firms with varying risk-return preferences pursue R&D, this conventional perspective continues to influence decision-making in both corporate strategy and economic policy. This paper questions the narrative by using a novel statistical framework that accounts for competitive strategy and environmental turbulences. Drawing on firm innovation data from the Community Innovation Survey (CIS), we apply *semiparametric regression for location and scale* to model both the mean and the variance of turnover growth as a function of the interaction between R&D intensity and environmental turbulence, across four common competitive strategy regimes. The findings reveal that for firms prioritizing price leadership across a broad product range, R&D is associated with reduced risk and minimal impact on average growth. Only for firms specifically focused on high quality or small product ranges, the results align with prior research, confirming the expected high-risk, high-return relationship associated with R&D.

## Introduction

The conversion of R&D investments into corporate growth – such as through the successful commercialization of new products – is uncertain. Due to the unpredictable nature of R&D outcomes [1], such investments are frequently regarded as high-risk endeavors [2,3]. Even when an R&D project is not inherently risky, the primary sources of uncertainty often lie in external factors, including shifting customer preferences, competitor innovations, and broader market dynamics [4,5]. This raises the question of whether R&D success can be effectively managed in highly turbulent environments [6–9].

Aside from that, innovation efforts are guided by diverse objectives – ranging from cost reduction to developing new products – which are shaped by a firm’s overarching competitive strategy. Distinct strategies imply different types of innovation. Yet, innovative activity is typically measured by R&D expenditures or patent grants, neither revealing any information about the nature or intent of the innovation itself. However, theoretical models suggest that the type of innovation is crucial to understanding the complex relationship between R&D, risk and return [10–14].

If R&D impacts differ by strategy, many past studies may overgeneralize by overlooking this key source of variation. This may raise concerns about the validity of findings that treat the effects of R&D as uniform across firms, without accounting for differences in strategic orientation. Such concerns are relevant not only for modeling innovation returns, but become especially critical when examining the relationship between R&D and corporate risk. Prior research suggests a generally positive association between R&D and corporate risk, such as the evidence provided by Coad & Rao [15] – though without considering differences in strategy. Moreover, most studies assume that any competitive strategy requires R&D [1,14]. Supposing that R&D is inherently risky, this implies that firms – regardless of their risk preferences – are compelled to accept a certain level of risk when aiming for competitive advantage [16]. However, this assumption has yet to be examined.

To fully understand the relationship, it is essential to also consider external factors. A substantial proportion of R&D risk and returns stems from the firm’s interaction with its environment [17,18]. Typically, firms first define their corporate and competitive strategy, and then allocate resources to achieve specific, predefined goals (though there are exceptions to this sequence, as noted by Kannan-Narasimhan & Lawrence [19]). In the standard sequence, the overall competitive strategy of a firm can be considered fixed in the short run, while the determination of R&D intensity remains variable during this period. The effects of R&D are still subject to moderation by environmental factors.

The present work integrates these elements into a single empirical model. We study how R&D intensity and industry dynamism as a form of turbulent environments are linked with firm growth and risk under different competitive strategy regimes. In doing so, we also explore to what extent R&D-related risk is shaped by environmental conditions under varying strategic orientations. Following a standard approach in the innovation literature, we use the mean and the variance of turnover growth rates to measure firm growth and risk [15,20]. The relationship to both firm growth and risk is modeled using an interaction effect composed of R&D intensity and environmental turbulence. This work additionally advances previous research by modeling both the mean and the variance of firm growth within the same model, using semiparametric regression, namely *Generalized Additive Models for location and scale* (GAMs) [21–23]. This approach allows for any functional form of the relation in the model. That is, the interaction effect of R&D intensity and environmental turbulence is a function, not restricted to have any a priori determined, specific form. This rather complex modelling framework is suggested since it is more suitable to capture the very dynamic structure of the relationship.

This paper proceeds with a literature review on innovation, competitive strategy, and environmental turbulence, which leads to the development of our research hypotheses. Next, we describe the dataset and introduce semiparametric location-scale regression as our novel statistical method. The findings and their interpretation are presented in a combined results and discussion section. The paper concludes by summarizing key insights and outlining limitations and implications for theory and practice.

### Literature review

Leading theoretical models of growth emphasize innovation as the key driver of both corporate and economic development [24–26]. Since the advent of large-scale firm-level microdata, a substantial body of literature has emerged aiming to enhance our understanding of innovation and its effects through empirical analysis. However, earlier findings on the average relationship were mixed and often failed to confirm the positive impact of innovation on corporate growth predicted by theory (e.g., [27,28]). More recent approaches could at least reveal a positive impact of R&D for anyway fast-growing firms, using rather sophisticated statistical methods (such as quantile regression instead of standard OLS regression) [20,29–32]. This growing body of evidence highlights the need for more advanced statistical procedures to explore the relationship. Starbuck [33] made the early prediction that “[the subject of corporate growth] is ready for – and badly needs – solid, systematic empirical research directed toward explicit hypotheses and utilizing sophisticated statistical methods” [33, p. 126].

While the measurement of corporate growth (or financial performance) is usually straight-forward (see, e.g., our data section), a central question is how to measure innovation. There are several indicators and proxies that are used in the literature, such as the number of patents filed or granted [34], the number of scientific publications [35], the number of new product announcements [36] or innovation-related job postings [37], trademark counts [38], the number of employees in R&D [39], and counts of innovation awards [40]. Other measures involve the number of patent citations as an indicator of the level of innovativeness [41] and the diversity of a firm’s patents across technological fields as an indicator of innovation diversity [42]. Additionally, when available, some studies incorporate survey responses to assess innovation activities and perceptions directly from firms [43]. However, the most common approaches involve the spending on R&D, either as absolute amount of total investment [44] or, more commonly, as a ratio to total sales (or turnover, used synonymously), referred to as ‘R&D intensity’ [45], which is also used in this study.

Studies have shown a significant statistical overlap between these indicators (and proxies), particularly in R&D-intensive, high-tech industries [40]. Moreover, previous contributions using different indicators for innovation consistently produced similar results, particularly with regard to the relationship between innovation and firm growth [20,46,47]. Hagedoorn & Cloodt [40] suggest that “the statistical overlap between these indicators is that strong that future research might also consider using any of these indicators”.

Yet, regardless of the proxy used to measure innovation activity, the relationship between firm growth and innovation remains contradictory. The anticipated strong, positive relationship between innovation and growth, as predicted by theoretical models, was not observed in firm-level data. The most cited explanation points to the increased corporate risk associated with innovation activities [29]. Although revenues can be huge, they are uncertain with low success rates and high costs of failure [16], leading to a greater variance of returns. The variance of returns or firm growth is the most commonly used measure of risk in the innovation literature [5,15,16,29,48,49]. The increased variance may obscure the true positive impact of R&D. Following a standard approach [15,20], In this study we measure the risk and returns associated with R&D by using the mean and variance of firm growth rates.

Despite of the substantial risk, many firms face pressure to engage in innovation in order to gain or maintain any competitive advantage [50–53]. Due to this innovation pressure, each competitive strategy is considered to be associated with substantial risk, “because an innovation strategy based on risk avoidance cannot be an option” [16, p. 30]. However, the innovation literature differentiates between innovation types, predicting entirely different impact mechanisms and risk implications [10]. Innovation types are usually either classified by their subject (e.g., product or process innovation) or based on their intended novelty (incremental or radical innovation). The most recognized performance- and risk-related differences are implied by the intended novelty of the innovation [54]. If a firm aims to improve a well-established product (or service) through incremental innovation, we assume that it faces a lower risk of failure than a firm that aims to disrupt an existing market with the introduction of a radical innovation. The type of innovation is usually chosen to be in line with the overall competitive strategy of the firm [55]. Not only the innovation portfolio itself, but also the resource allocation to the innovation portfolio is determined by the competitive strategy, which both revealed to have a significant influence on firm performance [17].

Competitive strategies are strategies that firms use to achieve a competitive advantage in their market [56]. Essentially, firms aim to compete either through lower prices or superior quality. Additionally, they consider the scope of their product range, targeting specific market segments. These two dimensions (*price/quality* and *broad/small range of products and services*) are the basis of the most widely recognized classification of competitive strategies into four generic categories: 1. Cost Leadership (CL), including firms aiming to be the lowest-cost producer industry-wide; 2. Differentiation Leadership (DL), covering firms that focus on unique products valued across the industry; 3. Cost Focus (CF), containing firms that target cost-efficiency in a specific market segment; and 4. Differentiation Focus (DF), offering unique products tailored to a particular market niche. Table 1 is a summary of the competitive strategies, based on Porter [57,58].

**Table 1:** Competitive strategies based on porter (1985), p. 11–15.

		Competitive advantage	
		Lower cost	Differentiation
**Competitive scope**	Broad target	1. Cost Leadership (CL)	2. Differentiation Leadership (DL)
	Narrow target	3. Cost Focus (CF)	4. Differentiation Focus (DF)

Since each of the competitive strategies presented in Table 1 can encompass a varying combination of innovation types [57–60], the impact of R&D within these regimes remains difficult to predict. However, certain tendencies can be identified based on the typical relationships between competitive strategies and innovation types outlined above.

Differentiation Leadership-oriented firms often rely on rather radical (product) innovation to create products (or services) with unique features (e.g., through breakthroughs in technology or entirely new product categories) [61,62]. Radical innovation efforts are typically regarded as involving high risks and potentially high returns [51,52]. As a result, Differentiation Leadership-oriented firms should face higher average growth rates and an increase in risk, on average. Accordingly, we predict the following:

*H1: In Differentiation Leadership-oriented firms, R&D has a strong positive impact on both the mean and the variance of firm growth.*

In contrast, Cost Leadership-oriented firms often engage in incremental (process) innovation to improve efficiency, typically aiming for economies of scale (e.g., by optimizing manufacturing processes or adopting lean practices, etc.) [63,64]. Because incremental innovation efforts are usually perceived as less risky yet also less profitable [2,10], firms adopting a Cost Leadership strategy are expected to experience slower growth and lower risks related to their innovation activities, compared to Differentiation Leadership-pursuing firms. Formally:

*H2: In Cost Leadership-oriented firms, R&D has a weak positive impact on both the mean and the variance of firm growth rates.*

The other competitive strategy categories, Cost Focus and Differentiation Focus, integrate elements of Cost Leadership and Differentiation Leadership, respectively, and are therefore partially associated with similar types of innovation. In niche segments, firms innovate more customer-centric. According to the customer-centric innovation literature [50,61], innovation types associated with these competitive strategies tend to be combinations of both radical and incremental innovations. Cost Focus- and Differentiation Focus-oriented firms are assumed to be rather focused on the demand of their specific customer group than on the product features alone. These firms hence strive to balance continuous improvements to meet evolving customer needs with breakthrough innovations to address the unmet demand of their specific market segment [61]. This is also why the ‘Differentiation Focus’ and ‘Cost Focus’ strategies are often consolidated under a single ‘Focus’ strategy in common frameworks, rather than being treated as distinct competitive strategies [57]. Since firms following ‘Focus’ strategies pursue a combination of innovation types, the impact of R&D is expected to be intermediate between those of the other two strategies. Accordingly, we predict the following:

*H3: In Cost Focus- and Differentiation Focus-oriented firms, R&D has a moderate positive impact on both the mean and variance of firm growth, with the effect lying between the strong impact in H1 and the weak impact in H2.*

However, a significant portion of R&D’s impact arises from its interaction with the firm’s environment [5,6]. Such environmental dynamism is usually subdivided into market turbulence, competition intensity, and the rate of technological change [8]. Changes in any of these factors can make innovation activity fruitless, even if R&D efforts are on track and successful.

A long tradition of research aims to understand the uncertainty driven by turbulences in the firm environment. There are distinct measures for market-, competition- and technology-driven turbulences, yet the most common method to measure environmental turbulences uses the variance of revenues within an industry as a proxy [54,65–78]. The revenue variance within an industry is influenced by all three dimensions: Market turbulences (e.g., through demand fluctuations) [79,80], competitive intensity (e.g., driving diversity of strategies and revenues) [57,71,81], and technological changes [70,82]. Beyond that, a greater variance may also indicate entirely exogenous shocks, such as macroeconomic or political events [83]. These facts make the revenue variance within industries an overarching and widely validated measure of environmental turbulences.

As discussed above, the existing literature posits that environmental factors account for a substantial portion of the risk associated with R&D. Hence, we expect R&D to be riskier in a turbulent environment and vice versa. However, the effect on average growth remains less predictable. High levels of environmental turbulence introduce greater uncertainty, making it difficult for firms to effectively plan and implement long-term strategies. This uncertainty may lead to erroneous decision-making and reduced average growth rates [84–88]. Conversely, environmental turbulence can create opportunities for firms that are agile and innovative. Such firms can leverage rapid changes to differentiate themselves, enter new markets, or develop novel products and services, potentially enhancing average growth rates [12,68,89,90]. Although both outcomes are possible, recent studies tend to assume a negative impact of environmental turbulence on average growth [91,92]. In particular, research on firm performance during periods of heavy environmental turbulence, such as institutional changes, indicates that the effect of environmental turbulence on average growth is predominantly negative [48].

The literature on both incremental and radical innovation suggests the same [5,7,8,16,52]. Since our hypotheses regarding the impact of R&D under different competitive strategies are grounded in these innovation types, we do not differentiate between strategies when predicting the impact of environmental turbulence. Instead, we expect turbulent environments to amplify the risk related to R&D across all competitive strategies while leading to a reduced positive impact of R&D on average growth. Accordingly, we formulate the following hypotheses:

*H4a: The relationship between R&D investment and the variance of firm growth is moderated by environmental turbulence, such that the positive effect of R&D on the variance of firm growth is stronger in highly turbulent environments, regardless of competitive strategy.*

*H4b: The relationship between R&D investment and average firm growth is moderated by environmental turbulence, such that the positive effect of R&D on average firm growth is weaker in highly turbulent environments, regardless of competitive strategy.*

To test our hypotheses, we constructed a firm-level data set by merging multiple sources. This integration provides the foundation for operationalizing key variables and conducting the hypothesis-driven analysis. In the following section, we describe the construction of our data set, the operationalization of variables, and the statistical modeling approach.

## Data and methods

### Data set

The sample used in our study is a merged data set, consisting of *two* widely used sources of financial and innovation firm microdata. The main basis of our analysis is the *Community Innovation Survey* (CIS) database, provided by the *European Statistical Office* (Eurostat). Eurostat is an administrative division of the European Union (EU) responsible for harmonizing and providing statistical information, including particularly for research purposes [93]. The series of surveys of which the CIS consists is executed by national statistical offices in the EU according to EU-wide standards. These standards aim to ensure a reliable survey methodology, representative samples and high-quality research data. After all, the CIS is the main source of information on innovation in Europe and contains detailed information on 7,647 very heterogeneous European firms represented from all industries. It is collected every two years, such that the total sample consists of two-year waves from 2008 to 2018.

Our second data source is the *Compustat Annual Industrial Database* (Compustat), which is provided by *Standard & Poor‘s Global Inc.* (S&P Global) [94]. S&P Global is primarily recognized as a trusted source of information in the financial sector [95] and Compustat is a primary source of market data widely used for analyzing industry dynamics [96,97].

We merged the two datasets using industrial classification codes of the European Union, namely the *Statistical Classification of Economic Activities in the European Community* (NACE). NACE uses four levels in its hierarchical system to differentiate economic areas. We used two-digit NACE codes to merge the CIS dataset with the Compustat data.

After merging the two datasets using NACE codes, our selection of industries to concentrate on in the analysis is finally obtained using the *General Industry Classification System* (GICS), according to Kile & Phillips [98], who extensively worked on the development of procedures to select samples of *high-tech* firms. We concentrate our analysis on the high-tech sector, where R&D expenses and innovation efforts are very high for a set of reasons: One reason is that previous studies have shown that the high-tech sector provides the most turbulent environment [95], such that the question we ask is most relevant in this sector. Another reason is the comparability with previous innovation studies, which do not include strategic dimensions in their empirical approaches. Lastly, we try to avoid sector aggregation issues in our empirical analysis [99]. Corresponding eight-digit GICS codes can be found in Table 2 of Kile & Phillips’ work [98]. As explained above, global industry dynamics are better captured in the Compustat database and based on previous research on global market connection [100], we assume that EU high-tech firms are affected by global market turbulences. We further exclude outliers (1% on each side) and firms which are not active in R&D, which leads to samples of 6,087 firms after cleaning; after filtering concerning competitive strategies (see below), the final sample size is 5,823.

**Table 2:** Competitive strategy question in the CIS.

	Degree of importance			
	High	Medium	Low	Not important
1. Focus on *low-price* (price leadership)	ο	ο	ο	ο
2. Focus on *high-quality* (quality leadership)	ο	ο	ο	ο
3. Focus on a *broad range* of goods or services	ο	ο	ο	ο
4. Focus on one or a *small number of key goods or services*	ο	ο	ο	ο

### Variables and statistical model

#### Main variables.

Firm growth is measured through turnover growth, which serves as the operational measure and is denoted by G. Turnover growth is defined as the growth rate in turnover between the beginning and the end of the survey wave of the CIS. We measure R&D using R&D intensity, a common approach that enhances comparability. R&D intensity is defined as the ratio of R&D expenses to microaggregated turnover and is denoted by RDI. Our competitive strategy variable is constructed using multiple questionnaire responses from firms regarding their competitive strategies. Specifically, we used survey answers aligned with the most common classification of competitive strategies, as detailed in the literature review. These categories are [57,58]: 1. Cost Leadership (CL), 2. Differentiation Leadership (DL), 3. Cost Focus (CF) and 4. Differentiation Focus (DF). The four categories differ in the focus on either *price* or *quality* and in the focus on either a *small range* or a *broad range* of key goods and services.

The question asked in the survey was: “*How important were the following strategies to the economic performance of your enterprise?*” Table 2 shows the response options to the competitive strategy question. We assigned firms to specific strategy subsamples based on their responses. A firm was included in a subsample for a specific strategy if it responded with at least “High” to both corresponding survey questions. For example, firms that responded with “High” to both “Focus on low-price” and “Focus on a broad range of goods or services” were categorized under the Cost Leadership subsample for further analysis. In this way, we use the main differentiation aspect of the competitive strategies to separate the firms, which is common practice [56–58,72]. Firms with hybrid responses, indicating mixed or unclear strategies, were excluded from the analysis to ensure clear comparisons between distinct strategic groups. The other groups are similarly distinguished based on their responses. DL firms prioritize both quality leadership (differentiation) and a broad product range, assigning “High” importance to both. CF firms emphasize cost leadership with a “High” focus on a narrow product range. DF firms similarly emphasize quality leadership but concentrate on a limited range of products, assigning “High” importance to both. The last main predictor variable, environmental turbulence, is denoted by TUR and is calculated using data from the Compustat industry dataset, as described earlier in the Dataset Section.

#### Control variables.

There is a substantial body of literature dedicated to possible effects of various control variables on corporate growth. In line with main works [101–103], we include firm size (measured by numbers of employees) and firm age (measured as the number of years since the firm’s founding) as control variables, since they have been shown to have (weak) effects in similar empirical settings. The use of this set of control variables has become a standard practice in corporate growth studies [20,28–32,46–48,96]. Due to the special panel structure of our sample, in each wave there are only two observations per firm that cannot be linked to other waves, which makes firm-fixed effects obsolete. Aside from that, earlier research has shown that firm growth is rather random on the longitudinal scale (see the large body of research in the tradition of “Gibrat’s Law” [104]) and that an omitted variable bias is very unlikely even in the cross-sectional view [105]. However, we cannot fully prevent endogeneity issues caused by reversed causality or firm heterogeneity [106].

#### Statistical model.

After subsampling firms that devote themselves mainly to one of the defined competitive strategies, we fit the following generalized additive models for location and scale with spline-based smooth functions (GAMs) [21–23]:


$$\begin{array}{c} h_{CL}\left(G_{i,t}^{CL}\right)=\beta_{0}^{CL}+f^{CL}\left({RDI}_{i,\;t-1}^{CL}\;|\;{TUR}_{i,t}\right)+\beta_{X}^{CL}X_{i,t-1}^{CL}+U_{i,t}^{CL}, \end{array}$$ ,(1)

$$\begin{array}{c} h_{DL}\left(G_{i,t}^{DL}\right)=\beta_{0}^{DL}+f^{DL}\left({RDI}_{i,\;t-1}^{DL}\;|\;{TUR}_{i,t}\right)+\beta_{X}^{DL}X_{i,t-1}^{DL}+U_{i,t}^{DL}, \end{array}$$ ,(2)

$$\begin{array}{c} h_{CF}\left(G_{i,t}^{CF}\right)=\beta_{0}^{CF}+f^{CF}\left({RDI}_{i,\;t-1}^{CF}\;|\;{TUR}_{i,t}\right)+\beta_{X}^{CF}X_{i,t-1}^{CF}+U_{i,t}^{CF}, \end{array}$$ ,(3)

$$h_{DF}\left(G_{i,t}^{DF}\right)=\beta_{0}^{DF}+f^{DF}\left({RDI}_{i,\;t-1}^{DF}\;|\;{TUR}_{i,t}\right)+\beta_{X}^{DF}X_{i,t-1}^{DF}+U_{i,t}^{DF}$$ (4)

where h() is a link function (see below), $G_{i,t}$ is turnover growth of firm i at year t, ${RDI}_{i,\;t}$ is R&D intensity, ${TUR}_{i,t}$ are industry turbulences (in the industry of firm i‘s main activity), $X_{i,t}$ is the set of control variables described above, f() is a non-parametric partial effect function used to model the functional form of the fitted predictor, made up by the interaction between ${RDI}_{i,\;t}$ and ${TUR}_{i,t}$, and $U_{i,t}$ is an error term.

Generalized Linear Models (GLM) [107] are frequently used to model non-normally distributed data when Ordinary Least Squares regression is not applicable due to non-fulfillment of the necessary assumptions. However, they allow the response variable only to be linked linearly with the predictors. Apart from their restriction concerning strict linearity in the relationship to predictors, they do not allow to model other summary statistics than the average turnover growth. Since in our case the variance as the measure of risk is a main aspect in the context of competitive strategies and R&D-related risk, we turn to using the more flexible GAM framework. By using GAMs, we can relax the linearity assumption while retaining additivity.

In our approach, each covariable is related to turnover growth via individual non-parametric functions f(). The generic form of a GAM can be written as


$$g\left(E\left(y_{i}\right)\right)=\beta_{0}+\sum_{j=1}^{p}f_{j}\left(x_{ij}\right).$$

In our analysis, special attention is devoted to these partial effect functions. They are approximated through basis function expansions (piecewise polynomials connected smoothly at knots). This function can be approximated using expansions of basis functions, i.e.,


$$f_{j}(x)=\sum_{k=1}^{K}b_{jk}B_{k}(x),$$

where $B_{k}$ denotes basis expansions with corresponding coefficients $b_{jk}$. These functions are further penalized to enforce specific properties, such as smoothness or sparsity through a smoothing parameter $\lambda_{j}$, by solving:


$$\min_{b}\sum_{i=1}^{n}{\left(G_{i,t}-b_{k}B_{k}({RDI}_{i,\;t-1}\;|\;{TUR}_{i,t})\right)}^{2}+\lambda {{(\Delta^{d}b}_{k})}^{2},$$

where $B_{k}()$ again denotes the basis expansions with corresponding coefficients $b_{k}$ and $\Delta^{d}$ denote the dth-order difference. We *use thin plate regression splines* [108] as smoothing functions because of their superior performance compared to alternative spline-based functions [109]. Smoothing parameters are automatically determined based on criterions such as the generalized cross validation score (GCV) [110] or (restricted) maximum likelihood (REML) [111].

In the models defined above, we estimate the interaction effect of R&D intensity and environmental turbulences as a spline-based smooth function in the 3-dimensional space. We refer to the effect of this interaction term, while accounting for the influence of other variables, as the partial (interaction) effect function. We test for the significance of the non-parametric interaction functions using a common Wald-type test [112]. This rather complex partial effect estimation was chosen due to the high number of observations available, which allows to deduce a more complex effect structure instead of just positive or negative coefficients. As well, the statistics literature suggests minimum sample sizes to employ semiparametric regression that are all clearly exceeded [23,113,114] and previous research has already refuted a possible linear relationship in a similar context [115]. We similarly argue that a better fit to the data can be accomplished by using non-parametric estimation compared to entirely parametric models. The marginal loss of interpretability that comes along with non-parametric models, i.e., by dropping interpretable, numeric regression coefficients, is not a considerable drawback in our context, since we are focused on general conclusions over precise numeric results, which are more beneficial to practitioners.

To incorporate both the mean and the variance in the same model within the GAM framework, we use a Gaussian location-scale model with identity-logb-link function for the fitting and GCV to determine the smoothness parameters in our spline-based partial effect functions. For fitting the model and for diagnostics, we used the R software package “gam” [116,117]. We also performed their model checking procedure (tests on adequate basis dimensions of the smooths, QQ plots, convergence tests, etc.; see, e.g., Wood [112]).

## Results and discussion

**Fig 1:**
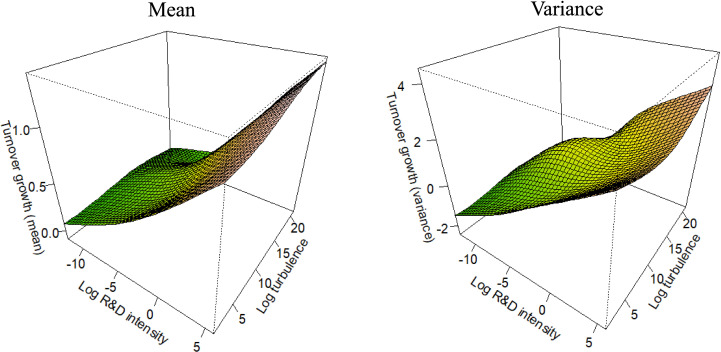
Partial effect functions for the differentiation leadership subsample (n = 1,542).

**Fig 2:**
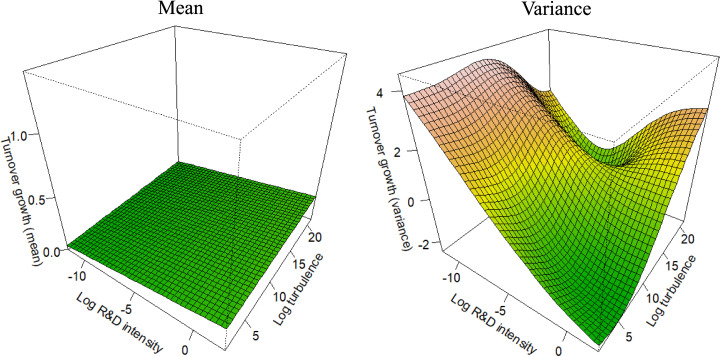
Partial effect functions for the cost leadership subsample (n = 1,324).

**Fig 3:**
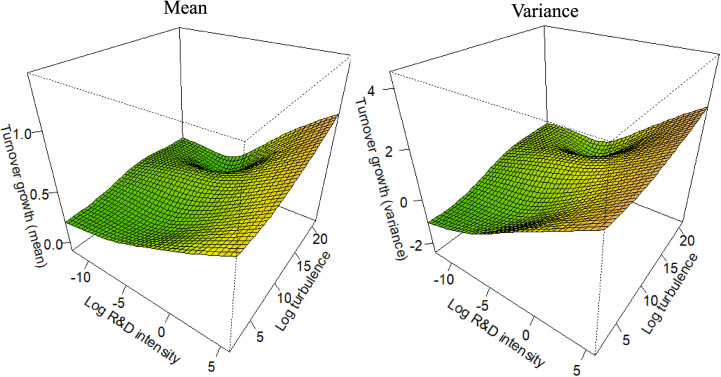
Partial effect functions for the Differentiation Focus subsample (n = 1,841).

**Fig 4:**
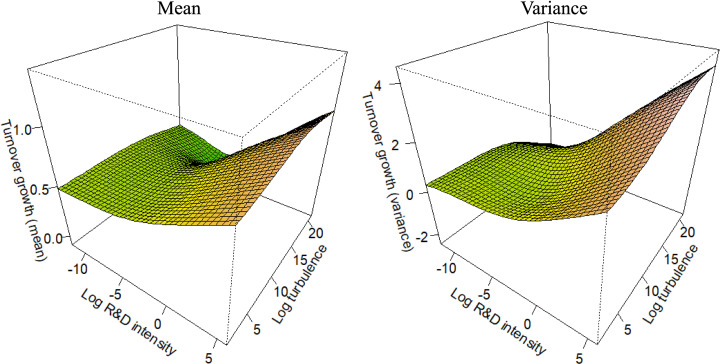
Partial effect functions for the Cost Focus subsample (n = 1,116).

For each competitive strategy subsample (as defined in Equations 1–4), Figs 1–4 depict the marginal effects of our primary predictor, with all other predictors held constant, to test the corresponding hypotheses. To enable the comparisons integral to the hypotheses, all figures use consistent axis scales. The non-linearity of the partial effect functions was significant at the 1% level across all settings, as determined by a Wald-like test for smooth components in GAMs [112]. The estimated degrees of freedom ranged from 6 to 7, unequivocally confirming the strong non-linear pattern in the functions.

Our first hypothesis predicts that, within the Differentiation Leadership (DL) subsample, R&D has a strong positive effect on both the mean and the variance of turnover growth. Fig 1 presents the corresponding partial interaction effect functions of the DL subsample, both for the mean turnover growth (left panel) and for the variance of turnover growth (right panel). Focusing on the R&D axis in Fig 1, we find that, for DL firms, R&D leads to an average increase in turnover growth while also amplifying its variance. This supports Hypothesis 1. Furthermore, these results align with the expectations of previous research by indicating the ‘classical’ high-risk, high-growth pattern [2,5,6,16,26]. Although risky, DL firms invest in R&D to strengthen their competitive advantage by enabling the creation of unique products.

Our second hypothesis predicts that, within the Cost Leadership (CL) subsample, R&D would have a weak but positive effect on both the mean and the variance of turnover growth. Fig 2 illustrates the partial interaction effect functions for CL firms. The left panel of Fig 2 shows the effect on mean turnover growth, while the right panel depicts the effect on the variance of turnover growth. The results indicate that R&D intensity has a minor positive association with average turnover growth, with an effect size less than 10% compared to the DL setting in *H1*. On the contrary, there is a strong negative association between R&D and the variance of turnover growth, which significantly contradicts Hypothesis 2. Overall, the results reveal that the impact of R&D in firms pursuing a Cost Leadership strategy differs from what we would anticipate based on the existing literature [2,5,6,26].

Our results indicate that, for CL firms, engaging in R&D yields only modest gains in average turnover growth, while refraining from R&D is associated with significantly higher turnover growth variance, implying greater uncertainty and risk. While prior research anticipated a lower impact of R&D on average turnover growth for CL firms compared to DL firms, the observed negative association between R&D and turnover growth variance within CL firms was not expected in this form. Previous research acknowledged that firms pursuing DL rely on uniqueness supported by extensive R&D, while CL firms rather focus on efficiency and cost control, which requires less extensive R&D. For CL firms, R&D primarily focuses on simplifying manufacturing and optimizing processes, as they are unable to command higher prices for their products. Excessive R&D expenditure, therefore, would contradict the fundamental principle of cost control in such firms [118]. Based on our findings, high R&D expenditures in CL firms can be understood as a strategic approach to risk management rather than a means of achieving (exceptional) growth. Our hypothesis was formulated based on the nature of the innovation types involved. However, these innovation types do not fully account for the underlying strategic goals associated with their implementation. To gain a clearer understanding of our findings, we may need to incorporate recent research on the R&D objectives of CL firms [119]. Typically, CL firms can leverage R&D to achieve profit gains either by enhancing cost efficiency or by expanding market share via lower prices, though only the latter would lead to turnover growth. Current research suggests that CL firms primarily use R&D to maximize profits through cost efficiency (whereas, when aiming for increased turnover, CL firms rather extend their product range) [120]. Moreover, unlike DL firms, the products or services offered by CL firms lack uniqueness, making it easier for competitors to gain market share if they fail to maintain cost leadership [121]. Therefore, not investing in R&D to maintain cost control can lead to increased uncertainty for CL firms. Overall, we find that the advantage of R&D in CL firms lies in risk management rather than in additional growth.

In *H3*, we hypothesize that, in both the Differentiation Focus (DF) and the Cost Focus (CF) context, R&D would have a positive effect on both the mean and the variance of firm growth. However, we anticipated that the effect would be weaker than in H1, but stronger than in H2. We assumed the innovation types of firms offering products or services tailored to a particular market niche to be combinations of radical and incremental innovation [50,61]. Figs 3 and 4 illustrate the partial interaction effect functions for firms employing a DF or a CF strategy, respectively. As in the previous figures, the left panel depicts the effect on mean turnover growth, while the right panel shows the effect on the variance of turnover growth*.* We examine the partial interaction effect functions focusing on the R&D axes in Figs 3 and 4. The results indicate a somewhat similar risk-return pattern compared to DL firms, though the effects are less pronounced. This supports *H3*. Moreover, since the DF and CF firms exhibit the typically assumed high-risk, high-growth pattern associated with R&D [2,5,6,16,26], our results remain consistent with previous research.

In H4a, we predicted that environmental turbulence moderates the positive effect of R&D on the variance of turnover growth, amplifying the magnitude of the effect. Additionally, we expected that this moderating influence is consistent across different competitive strategies. To test H4a, we analyze the partial interaction effect functions of the competitive strategy subsamples in Figs 1–4, focusing on the right panels (which depict the effect on the variance) and the turbulence axes. The results show that in each competitive strategy subsample, higher environmental turbulence is associated with increased turnover growth variance, with no substantial variation across the subsamples. However, the effect is not apparent at low turbulence levels in DL firms. Moreover, the effect is more pronounced in DF and CF firms than in DL and CL firms, indicating that focusing on a small range of products (or services) is related to increased uncertainty in turbulent environments. Overall, the results support Hypothesis 4a.

In *H4b*, we predicted that environmental turbulence moderates the positive effect of R&D on the mean of turnover growth, reducing its impact. Furthermore, our prediction was that this moderating effect is consistent across different competitive strategies. To test *H4b*, we analyze the partial interaction effect functions of the competitive strategy subsamples in Figs 1–4, but focusing on the left panel which depicts the effect on the mean. The results reveal that environmental turbulence is associated with higher average turnover growth in each competitive strategy subsample, which contradicts Hypothesis 4b. However, this finding aligns with prior research discussed in the literature review [12,68,90,91], suggesting that turbulent environments provide opportunities for agile and innovative firms. Additionally, previous studies indicate that high profits are achievable only in dynamic environments, resulting in positive turnover outliers that increase the average turnover growth [90].

In summary, the results do not support *H4b*. While we hypothesized a negative moderating effect, the data reveal a positive one – consistent across all strategy types, yet notably weaker for the CL subsample. Furthermore, although *H4a* predicted a positive effect on the mean and *H4b* a negative effect on the variance, both effects turn out to be positive. This finding aligns with prior research suggesting that turbulent environments compel firms to innovate, thereby increasing the likelihood of both substantial gains and losses in growth [71,72,79].

## Conclusions

The present work investigates how R&D intensity and environmental turbulence are linked with firm growth and risk under different competitive strategy regimes. Using data from the largest European innovation survey, the Community Innovation Survey (CIS), we applied a semiparametric location-scale model that includes an interaction between R&D intensity and environmental turbulence. Methodologically, this study responds to calls in the literature for more advanced statistical approaches to better capture the complex relationship between R&D, firm growth, and risk. The semiparametric location-scale model allows us to flexibly estimate the functional form of the relationship between R&D and both firm growth and risk, rather than assuming it a priori. At the same time, to ensure comparability and robustness, our operationalization of key constructs – such as R&D intensity and environmental turbulence – follows widely accepted practices in the field.

Our findings contribute to ongoing debates on the heterogeneous impact of R&D. Frist, we show that the effects of R&D vary substantially depending on a firm’s competitive strategy – challenging prior research that implicitly assumed the impact of R&D to be similar across competitive strategies [20,29–32]. Second, we demonstrate that R&D is not necessarily a high-risk endeavor in all contexts, as suggested by prior research [2,5,6,16,26]. Specifically, for firms pursuing a Cost Leadership strategy in relatively stable environments, investing in R&D may involve lower risk than refraining from such investment. This suggests a shift in perspective: R&D can, under certain strategic and environmental conditions, act as a form of risk mitigation rather than as a high-risk innovation initiative. These insights help explain why previous studies often failed to identify consistent positive effects of R&D on average growth [27,28], despite strong theoretical expectations [25,26]. Our results suggest that the value of R&D is context-dependent and may stem not only from its growth potential but also from its stabilizing effect on firm performance.

From a strategic management perspective, our findings caution against generic assumptions about the role of R&D in firm performance. Rather than treating R&D as inherently risky or universally growth-inducing, firms should evaluate its relevance in light of their competitive strategy and environmental context. This perspective enables more informed decision-making and may help avoid costly misalignments between innovation investments and strategic positioning.

From an innovation policy perspective, our findings highlight the shortcomings of generic R&D support. Policy frameworks should consider both the strategic orientation of firms and the environments in which they operate. For example, while Cost Leadership firms may benefit from R&D in terms of reduced risk, they are unlikely to contribute substantially to aggregate growth. Conversely, firms pursuing quality-focused or niche strategies appear more likely to translate R&D investments into growth, although at higher risk.

We also acknowledge several limitations and areas for future research. First, some firms do not fit neatly into the strategic categories used here or pursue hybrid strategies. Second, a more granular industry classification (e.g., using 4-digit NACE codes) could improve precision. Third, while we captured turbulence via industry-level sales dynamics, future work could disaggregate this measure to reflect distinct types of environmental change, such as technological, customer-related, or competitive turbulence. Finally, we assumed stable strategy classifications over two-year intervals; future longitudinal research could explore dynamic strategic shifts and their interaction with innovation outcomes. As highlighted in the ‘Methods’ section, ensuring the causal directions suggested in our study would also be a valuable avenue for further study.
